# The impact of the incorporation of a feasible postoperative mortality model at the Post-Anaesthestic Care Unit (PACU) on postoperative clinical deterioration: A pragmatic trial with 5,353 patients

**DOI:** 10.1371/journal.pone.0257941

**Published:** 2021-11-15

**Authors:** Claudia de Souza Gutierrez, Katia Bottega, Stela Maris de Jezus Castro, Gabriela Leal Gravina, Eduardo Kohls Toralles, Otávio Ritter Silveira Martins, Wolnei Caumo, Luciana Cadore Stefani

**Affiliations:** 1 Postgraduate Program in Medical Sciences, Universidade Federal do Rio Grande do Sul (UFRGS), Porto Alegre, Brazil; 2 Anaesthesia and Perioperative Medicine Service, Hospital de Clínicas de Porto Alegre (HCPA), Porto Alegre, Brazil; 3 Department of Statistics, UFRGS, Porto Alegre, Brazil; 4 School of Medicine, UFRGS, Porto Alegre, Brazil; 5 Department of Surgery, School of Medicine, UFRGS, Porto Alegre, Brazil; 6 Laboratory of Pain & Neuromodulation, School of Medicine, UFRGS, Porto Alegre, Brazil; Fondazione IRCCS Istituto Nazionale dei Tumori, ITALY

## Abstract

**Background:**

Practical use of risk predictive tools and the assessment of their impact on outcome reduction is still a challenge. This pragmatic study of quality improvement (QI) describes the preoperative adoption of a customised postoperative death probability model (SAMPE model) and the evaluation of the impact of a Postoperative Anaesthetic Care Unit (PACU) pathway on the clinical deterioration of high-risk surgical patients.

**Methods:**

A prospective cohort of 2,533 surgical patients compared with 2,820 historical controls after the adoption of a quality improvement (QI) intervention. We carried out quick postoperative high-risk pathways at PACU when the probability of postoperative death exceeded 5%. As outcome measures, we used the number of rapid response team (RRT) calls within 7 and 30 postoperative days, in-hospital mortality, and non-planned Intensive Care Unit (ICU) admission.

**Results:**

Not only did the QI succeed in the implementation of a customised risk stratification model, but it also diminished the postoperative deterioration evaluated by RRT calls on very high-risk patients within 30 postoperative days (from 23% before to 14% after the intervention, p = 0.05). We achieved no survival benefits or reduction of non-planned ICU. The small group of high-risk patients (13% of the total) accounted for the highest proportion of RRT calls and postoperative death.

**Conclusion:**

Employing a risk predictive tool to guide immediate postoperative care may influence postoperative deterioration. It encouraged the design of pragmatic trials focused on feasible, low-technology, and long-term interventions that can be adapted to diverse health systems, especially those that demand more accurate decision making and ask for full engagement in the control of postoperative morbi-mortality.

## Introduction

Postoperative complications are under recognized cause of morbimortality, being higher in a particular vulnerable group of high-risk patients undergoing elective and emergency surgeries [1,2]. The incidence of these adverse postoperative outcomes varies considerably between hospitals in diverse international health contexts [3–5].

To improve surgical patient assistance the perioperative re-engineering, optimization of processes by timely engagement of patients, caregivers and structure have been proposed [5,6]. However, local solutions adapted to the singularities of each health system should be considered.

Working with the population of a Brazilian university hospital, we developed the SAMPE Model to predict in-hospital deaths within 30 days after surgery. Showing the characteristics of an ideal risk model, this tool is parsimonious, highly accurate, and consists of four variables collected preoperatively: ASA-Physical Status (ASA-PS) classification, patient age, severity of surgery and nature of surgery (elective versus urgent) [7]. Following the definition of the Royal College of Surgeons, we considered those with predicted mortality above 5% as high-risk patients. To facilitate communication and the planning of care processes, we adopted four risk classes according to predicted mortality risk: Class I (p < 2%); Class II (2–5%); Class III (5% - 10%); and Class IV (≥ 10%) [7,8].

Identifying high-risk patients is not enough, though. It can be considered the first step to build a “system of safety” composed of horizontal care, continuous reassessment of risks, prediction, and planning [9]. Considering the limited and unequal distribution of Intensive Care Units (ICU) resources and the wide variability of allocation criteria, adopting local and pragmatic solutions such as the proactive care to vulnerable, high-risk patients, delivered in less intensive environments, might be an alternative to the high-dependency units (HDU) or ICU [10].

Thus, we implemented a quality improvement program at the Post-Anaesthetic Care Unit (PACU) consisting of improved identification of high-risk patients, adoption of a distinct discharge checklist, and optimization of handover. This is a pragmatic study aimed to describe the preoperative adoption of a customised postoperative death probability model and to evaluate the impact of incorporating a PACU pathway for high-risk surgical patients. We hypothesised that we might be able to reduce clinical deterioration in high-risk patients by stratifying them utilising an instrument calibrated to the population they are inserted in and individualising postoperative care through the implementation of an immediate postoperative high-risk surgical pathway.

## Material and methods

We conducted a quasi-experimental study comparing two groups: before and after the adoption of a quality improvement (QI) intervention to incorporate the preoperative risk stratification and the adoption of a PACU high-risk surgical pathway into clinical practice. The Ethical Committee for Postgraduate and Research Groups at the Hospital de Clínicas de Porto Alegre (HCPA)–Brazil (project number 2016–0229) provided ethical approval for this study. Written informed consent was not required, given the dataset includes only those documented as part of routine clinical care and that the data is collected in a fully anonymised format. The authors signed a confidentiality agreement to assess the hospital database.

### Data source and study population

We conducted this study at HCPA, a quaternary-level health centre in southern Brazil. We collected data from surgical patients over 16 years of age, referred to recovery rooms immediately after their surgeries, in day shifts between 07:00 am and 07:00 pm, from August 2016 to January 2017. We excluded from the analysis patients who underwent cardiac, obstetric, and outpatient procedures sent to ICU after their surgeries. When more than one surgical procedure was performed during the same hospital admission, we included only the major one. A team of trained researchers from the Anaesthesia and Perioperative Medicine Service collected data using information from the medical records and from the integrated information system of the hospital. The controls were patients who underwent surgeries at the same institution and had received usual care six months before the beginning of the intervention.

### Procedures

The key feature of the QI intervention was to introduce, in clinical practice, the risk assessment and triage to identify high-risk patients followed by an effective use of risk information to change assistance. The present study reveals the changes made in clinical processes to attempt a PACU pathway for high-risk surgical patients.

Postoperative HDU are not available in our hospital, and ICU beds are scarce and prioritised for neurosurgical care or patients with intraoperative events or instabilities (haemodynamic or mechanical ventilation). The PACU, for instance, is a highly demanded unit that handles not only low and high complexity patients but also many features of critical care therapies such as inotropic as well as short periods of invasive and non-invasive ventilation. The development of risk assessment and the improvement of the PACU pathway are grounded in the need for an individualised assistance and a discharge ward plan for the vulnerable ones.

The QI’s key features of an immediate postoperative care pathway for high-risk patients were:

1. A 12-week staff training to debate the problems of postoperative morbimortality of high-risk surgical patients in the public health system and interactive meetings to identify barriers to and solutions for the implementation of the new pathway.
2. Support teams to implement the calculation of individual predicted probability of death and respective risk class according to the SAMPE Model, a risk prediction tool for in-hospital postoperative deaths, developed in the same institution. We categorised patients into four risk classes according to the predicted probability of death: Class I (p < 2%); Class II (2% ≤ p < 5%); Class III (5% ≤ p < 10%); and Class IV (≥ 10%).
3. Implementation of visual identification of patients through coloured cards placed above the headboards of the beds in the PACU, being one colour for each of the risk classes.
4. Adoption of a distinctive discharge-to-ward checklist for patients classified as high-risk and very high-risk (classes III and IV). The recommended discharge checklist for high-risk patients included assessment of fluid balance and urine output, verification of laboratory tests and the need for new ones, review of medical prescriptions, and a written discharge plan, in addition to the standard checklist employed at the institution, based on the Aldrete-Kroulik and White scales (S1 and S2 Tables and S1 Fig).

**Fig 1:**
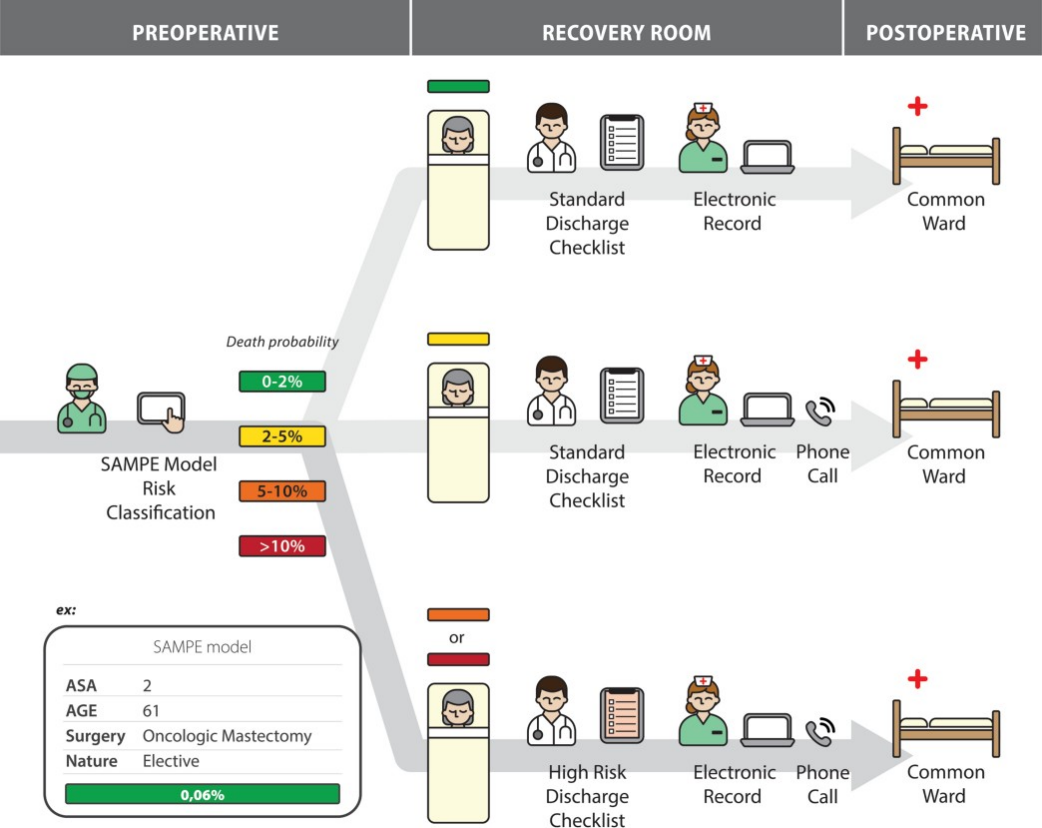
Schematic representation of the care flow of surgical patients after implementation of the SAMPE Model.

Discharge of high-risk patients by the PACU nurse included a report on the electronic medical record and a handover phone call to the ordinary ward nurse. Additionally, for low-risk patients (probability of death < 2%) with uncomplicated postoperative courses, the nurse handover process was simplified by dispensing with the need for handover phone calls from the PACU nurse to the ward nurse (Fig 1). After discharged from the recovery room, all patients received the usual care in the wards.

### Outcomes

To compare the clinical deterioration for similar patients allocated to different standards of care as part of their routine treatment, using a large dataset, we chose the accessible outcome of postoperative Rapid Response Team (RRT) calls within 48 hours, 7 and 30 postoperative days. The secondary outcome was in-hospital death within 30 days., and non-planned postoperative ICU admission. We conditioned RRT activation on the occurrence of the following triggers indicative of clinical deterioration: airway at risk (need for ventilatory support); respiratory rate < 8 or > 35 breaths/min; oxygen saturation < 90% (with or without the use of supplemental oxygen therapy); heart rate < 40 or > 140 beats/min; systolic blood pressure < 90 mmHg; decrease in the Glasgow coma scale ≥ 2 points; prolonged (>5 minutes) or repeated seizures. Patients were monitored for up to 30 postoperative days for the in-hospital occurrence of the outcomes. Those that were discharged before were considered free of complication.

### Statistical analysis

We used a “before and after” analysis to assess the impact of the incorporation of the PACU postoperative pathway. The outcomes were the occurrence of RRT calls within 48 hours, 7 and 30 postoperative days. From the hospital data provided by the RRT team, we estimated that between 8 and 10% of surgical patients would require RRT calls within 30 days, regardless of risk class.

With a 5% significance level and statistical power of 90%, we used a sample composed of 1,615 patients in each group to detect an RRT reduction of 25% in the intervention group compared to the usual care group. We employed a robust variance logistic regression model (Poisson model) to compare the proportion of RRT calls, deaths, and non-planned postoperative ICU between groups and the SAMPE Model risk classes.

The Poisson regression model with robust variance estimates relative risk or prevalence ratio as measure of association. It has de advantage to overcome the baseline disparities of outcome risks between groups. In our study the risk of outcome clearly differed from one SAMPE category to another. Poisson regression was used to compare the proportion of RRT calls between the two groups (before and after the quality improvement intervention) and to analyze the effect of interaction between the independent variables (SAMPE risk class and group). However, to avoid the overestimation of the RR error a robust error variance—a sandwich estimation—was used. We analysed data using the SAS Studio software version 9.4.

## Results

**Fig 2:**
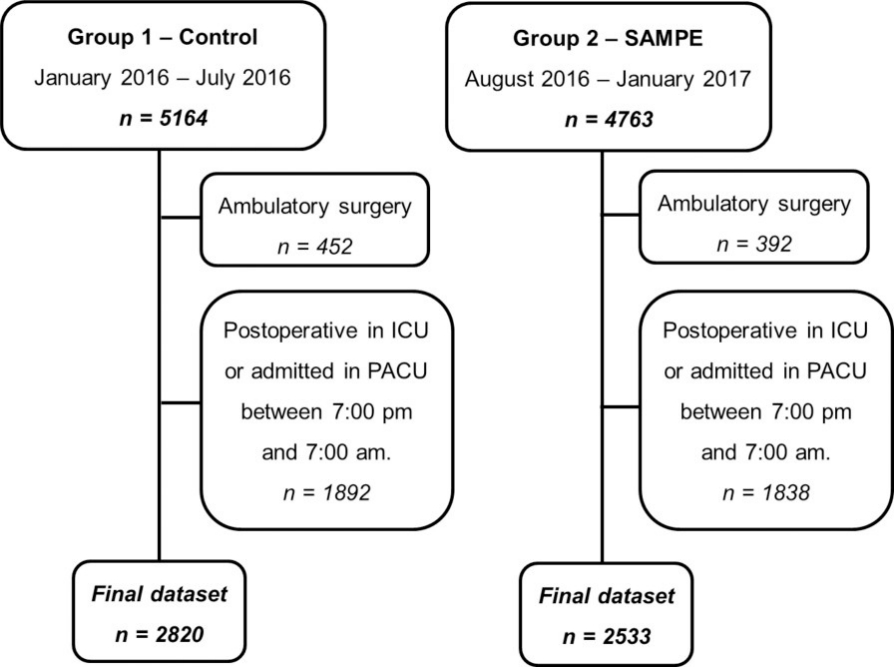
Trial diagram depicting the analysis of the SAMPE Model implementation. ICU, Intensive Care Unit; PACU, Postoperative Anaesthetic Care Unit.

We analysed data from a final retrospective cohort of 2,820 patients and a prospective one of 2,533, including 132 RRT calls within 48 hours (2.50%) and 234 within 30 days (4.37%). From August 1, 2016 to January 31, 2017, 2,533 patients were stratified according to the SAMPE Model during pre-anaesthetic evaluation. They were submitted to the immediate postoperative high-risk pathway when the probability of death exceeded 5%. The control group was a retrospective cohort of 2,820 patients that had received usual care with no risk stratification from January to July 2016. Fig 2 shows the potential inclusion in each group along with the individuals included in the final analysis of the primary endpoint. Table 1 shows the population characteristics of the intervention and control groups.

**Table 1:** Characteristics of Group 1 (control) and Group 2 (intervention).

	Control Group (n = 2820)	Intervention Group (n = 2533)	p-value*
**Age (years), mean ± SD**	54.02 ± 16.67	53.91 ± 16.57	
**Age (years)**			
16–35	450 (16%)	418 (16.5%)	0.58
36–55	931 (33%)	845 (33.4%)	0.78
56–75	1178 (41.8%)	1048 (41.4%)	0.76
> 76	261 (9.3%)	221 (8.7%)	0.49
**Gender**			
Male	1178 (41.8%)	1061 (41.9%)	0.93
Female	1642 (58.2%)	1472 (58.1%)	
**ASA-PS**			
1	556 (19.7%)	462 (18.2%)	0.16
2	1616 (57.3%)	1451 (57.3%)	0.98
3	624 (22.1%)	590 (23.3%)	0.32
4	24 (0.9%)	30 (1.2%)	0.22
**Surgery type**			
Elective	2246 (79.6%)	2095 (82.7%)	0.004
Emergency	574 (20.4%)	438 (17.3%)	
**Surgical severity**			
Minor	822 (29.1%)	820 (32.4%)	0.01
Intermediate	1270 (45%)	1103 (43.5%)	0.27
Major	728 (25.8%)	610 (24.1%)	0.14
**SAMPE risk class** ^**#**^			
I	2181 (77.3%)	1938 (76.5%)	0.47
II	270 (9.5%)	269 (10.6%)	0.20
III	225 (7.9%)	196 (7.7%)	0.74
IV	144 (5.1%)	130 (5.1%)	0.96
**Surgical specialty**			
General and digestive surgery	396 (14.0%)	333 (13.14%)	0.33
Vascular	256 (9.07%)	188 (7.42%)	0.02
Urology	442 (15.67%)	476 (18.79%)	0.002
Orthopaedics	310 (10.99%)	300 (11.84%)	0.32
Neurosurgery and spinal	62 (2.19%)	52 (2.05%)	0.71
Gynaecology and breast surgery	288 (10.21%)	252 (9.94%)	0.74
Colorectal	105 (3.72%)	90 (3.55%)	0.73
Upper gastrointestinal and hepatobiliary	585 (20.74%)	501 (19.77%)	0.38
Thoracic surgery	105 (3.72%)	78 (3.07%)	0.19
Ear, nose and throat	210 (7.44%)	197 (7.77%)	0.64
Plastic surgery	46 (1.63%)	46 (1.81%)	0.60
Maxillofacial	15 (0.53%)	20 (0.78%)	0.24

*Chi square test.

# Stefani L, Gutierrez C, Castro S, et al. Derivation and validation of a preoperative risk model for postoperative mortality (SAMPE model): An approach to care stratification. PLOS ONE. 2017;12(10):e0187122. doi:10.1371/journal.pone.0187122.

ASA-PS, ASA-Physical Status; SD, Standard Deviation.

### Prediction and prevention of postoperative deterioration

The majority of RRT calls occurred within the first three postoperative days (71.48%, n = 168). The main triggers for RRT calls were oxygen saturation < 90% (38.88%, n = 91) and systolic blood pressure < 90 mmHg (34.61%, n = 81). In 29.63% (n = 67) of patients, sepsis was suspected.

We did not find any significant differences in the total proportion of RRT calls between the control and intervention groups within 48 hours, 7 or 30 postoperative days. However, when SAMPE risk classes were analysed separately, we found out as an effect of the intervention, a reduction in RRT calls for class IV (probability of death > 10%) within 30 days. There was an interaction between group and risk class (p< 0.01) and a difference between risk classes regardless of group (p< 0.001). We observed an increase in the proportion of RRT calls in class II patients and a reduction in classes III and IV after implementation of the QI at the PACU. We summarised the results in Table 2. The SAMPE risk class was an independent predictor of RRT calls within 48 hours, regardless of group (p< 0.001). A significant difference was observed for risk class I compared to the other risk classes (p< 0.05). No significant difference was observed between groups in in-hospital death, non-planned ICU admission or duration of hospital stay with usual care *vs* QI intervention (Table 3).

**Table 2:** Rapid Response Team calls at 30 postoperative days before and after the quality improvement program.

	Total Sample		Control Group		Intervention Group		
SAMPE Class	Overall	RRT call	Overall	RRT call	Overall	RRT call	p-value*
**I**	4119	102 (2.47%)	2181	47 (2.15%)	1938	55 (2.84%)	0.16
**II**	539	37 (6.86%)	270	12 (4.44%)	269	25 (9.29%)	0.02
**III**	421	42 (9.97%)	225	25 (11.11%)	196	17 (8.67%)	0.40
**IV**	274	53 (19.34%)	144	34 (23.61%)	130	19 (14.62%)	0.05
**Total**	5353	234 (4.37%)	2820	118 (4.18%)	2533	116 (4.57%)	0.60

*Poisson Regression Model. RRT, Rapid Response Team.

**Table 3:** Patient outcomes—in-hospital mortality, Unplanned ICU admission, RRT calls within 7 and 30 postoperative days, before and after the quality improvement program.

	Usual Care (n = 2820)	Quality Improvement (n = 2533)	RR (CI) (Quality Improvement *vs* Usual Care)*	p-value*
**In-hospital all-cause mortality**	49/2771 (1.74%)	41/2492 (1.62%)	0.71 (0.46–1.10)	0.13
**Unplanned ICU**	76/2744 (2.70%)	83/2450 (3.28%)	1.22 (0.89–1.65)	0.20
**Duration of Hospital Stay** ^§^	2.07 (0.98–4.82)	2.07 (0.97–4.23)		0.32
**RRT 7 days Class I**	43/2138 (1.97%)	54/1884 (2.79%)	1.41 (0.95–2.10)	0.08
**RRT 7 days Class II**	10/260 (3.70%)	20/249 (7.43%)	2.01 (0.96–4.21)	0.06
**RRT 7 days Class III**	23/202 (10.22%)	17/179 (8.67)	0.85 (0.49–1.54)	0.59
**RRT 7days Class IV**	27/117 (18.75%)	14/116 (10.77%)	0.57 (0.32–1.05)	0.07
**RRT 30 days Class I**	47/2134 (2.15%)	55/1883 (2.84%)	1.35 (0.92–1.98)	0.12
**RRT 30 days Class II**	12/258 (4.44%)	25/244 (9.29%)	2.10 (1.08–4.11)	0.02
**RRT 30 days Class III**	25/200 (11.11%)	17/179 (8.67%)	0.76 (0.42–1.36)	0.35
**RRT 30 days Class IV**	34/110 (23.61%)	19/111 (14.62%)	0.61 (0.37–1.02)	0.06

*Poisson Regression Model.

^§^Median (IQR), n (%), or RR with 95% CI. CI, Confidence Interval; ICU, Intensive Care Unit; RR, Relative Risk; RRT, Rapid Response Team.

### Postoperative mortality

In-hospital mortality in the total sample was 1.68% (n = 90). We found no differences between the control and intervention groups in in-hospital postoperative mortality within 30 days. The mortality rate of patients treated by the RRT team was 10 times higher, 19.57% (n = 46). Among patients who died, 53% (n = 48) underwent major surgery; 54% (n = 49) of the surgeries were urgent, and 78% (n = 71) of the patients were classified as ASA 3 or 4. Table 4 shows the mortality by SAMPE risk class, with Class III and IV high-risk patients (13% of the total) accounting for 66% of all deaths. The main cause of the deaths was sepsis, followed by advanced cancer. Frequencies by death causes for the 90 cases are available in S2 Fig.

**Table 4:** Mortality by SAMPE Model risk class.

SAMPE risk class	Total sample	Deaths	% deaths within total sample	% within deaths of all deaths
**I—Probability of death: < 2%**	4119	15	0.4%	16.7%
**II—Probability of death: between 2 and 5%**	540	16	3%	17.8%
**III—Probability of death: between 5 and 10%**	403	17	4%	18.9%
**IV—Probability of death: ≥ 10%**	274	42	15.3%	46.7%

## Discussion

The main finding of this study was that a quality improvement program in the immediate postoperative care, a PACU pathway, had a modest impact on very high-risk patients regarding postoperative deterioration evaluated by RRT calls within 30 postoperative days. This intervention did not benefit the survival of patients or the reduction of non-planned ICU. Surprisingly, we found an increase in the number of RRT calls for intermediate-risk class patients. Moreover, we confirmed that the small group of high-risk patients (Class III-IV, 13% of the total) accounted for a higher proportion of RRT calls (40%) and 2/3 of postoperative deaths.

We described the implementation of an achievable postoperative pathway using a customised risk stratification tool to quantify risks for patients, providing differentiated care and discharge plans for those considered more vulnerable to postoperative complications.

The quality improvement program proved successful in the inclusion of a consistent, statistically oriented stratification tool, the SAMPE risk model, in the decision making of postoperative assistance. The decision tree has a great component of subjectivity because it is grounded by many difficult-to-measure factors such as individual preferences, cognitive biases, emotions, and previous life experiences [11,12], We intended to provide a template based in a customized toll to improve the judgment accuracy and to support a clinical pathway to increase patient safety. This tool is particularly useful when we consider the singularities of the Brazilian public health system where worsened conditions of diseases and unequal ICU resources compound the surgical scenario. The intervention was, however, restricted to PACU and, after discharge, all patients received standard ward care. We did not evaluate patients admitted to critical units. The brief intervention restricted to PACU and the handover processes might have contributed to the absence of expressive results. Also, the focus on giving better care to high-risk patients at the PACU might have led to unwanted suboptimal care of low-risk patients.

We should, also, be cautious when interpreting the RRT calls as a measure of the quality of assistance. Although the overall burden of postoperative morbidity can be associated with the increase in RRT calls, it may also express better early recognition of postoperative complications and represent supporting postoperative rescue and proper scaling up of care [13–15]. Furthermore, there is, nowadays, data suggesting that adverse events soon after PACU discharge are commonly undetected and unmanaged and can lead to serious adverse events in moderate-risk surgical patients under standard postoperative ward care [16]. This might signalise that no matter how good the trans and immediate postoperative care is, an intensification of monitoring is desirable at least in 24–48 hours. Alternatives to high-dependency and Intensive Care Units such as overnight at PACU or specialist wards in delivering postoperative care following high-risk surgery, should be pointed out [17].

Our study has some limitations. First, its design comprehended historical group comparison, which did not allow for the control of all potential confounding variables or other unmeasured changes in patient care during the studied period that may have affected clinical outcomes. Secondly, our sample included a small number of patients in the higher-risk categories (13%) compared to the lower risk ones, and a smaller-than-expected number of RRT calls in sample size calculation, leading to an underpowered analysis. Excluding surgeries performed at weekends or night most probably contributed to the high proportion of low-risk patients in our sample. Third, we used a large dataset from our management information system to compare outcomes for similar patients submitted to different standards of care as part of their routine treatment. This approach led to a poor understanding of each patient and did not enable a robust baseline risk adjustment and more granular outcome measures such as perioperative complications [10]. By restricting our analysis to the mortality rate, we were unable to distinguish between potentially preventable deaths and inevitable ones [18,19]. Mortality in the group of patients treated by the RRT in our study was close to 20%, attesting to the reliability of this index as an indicator of clinical deterioration [18–20]. Fourthly, the SAMPE model has not been externally validated, but its accuracy was recently confirmed, and a similar model was developed (Ex-Care model) and compared to classical risk tools [21].

Few studies have addressed the implementation strategy and the impacts of using individual stratification to guide surgical decision making and a coordinated approach of perioperative caregivers before, during and after surgery [22,23]. Recent results of the EPOCH study, a large-scale national QI program aimed at implementing a care pathway for patients undergoing emergency abdominal surgery, showed no survival benefits. The results were attributed to the variable fidelity of hospitals when implementing and prioritising pathway components, and to the time required to achieve effective change. The study suggests that QI programs ought to implement fewer, more discrete changes and ensure that leadership teams have adequate time to perform sustained improvements in patient care [24].

### Study contributions and future perspectives: Ex-Care project (Extended Care in High-Risk Surgical Patients)

Considering that high-risk surgical populations account for the highest number of postoperative complications and deaths and that postoperative care should ideally be individualised, we must extend such care beyond the boundaries of the operating room. For patients, the surgery journey begins when the surgery is decided upon in primary care and ends weeks or months after the procedure [25]. Measures proposed to address the challenge of improving patient experience and population health while reducing costs include risk-adapted postoperative management focused on the improvement of handover processes, better decisions concerning postoperative allocation, and well-designed ward processes aiming at preventing failure to rescue [26]. Accordingly, our future goal is the evaluation of an extended pathway (Ex-Care project)—Extended Care for High-Risk Surgical Patient—Clinical trials (https://clinicaltrials.gov/ct2/show/NCT04187664) based on multi-professional actions and process improvements that include risk communication, intensification of perioperative visits and close monitoring of vital signs of high-risk surgical patients for 48 hours during the postoperative period in the ward. We believe that the adoption of perioperative care pathways compatible with individual risks may improve patient experience and produce better outcomes (S3 Fig).

## Conclusions

This study described the implementation of a customized risk stratification tool and the limited impact of an immediate postoperative pathway aimed at high-risk surgical patients. On the other hand, it provided a template to start culture change and grounded the design of pragmatic trials focused on feasible interventions for high-risk patients in an inequity-based environment that demands more accurate decision makings and clamours for engagement against postoperative morbi-mortality.

## Supporting information

S1 Fig Quality improvement strategies. (TIF)

S2 Fig Frequencies of death causes (n = 90). (TIF)

S3 Fig Schematic representation of the care flow of high-risk surgical patients (Ex-Care pathway). (TIF)

S1 Table Standard PACU checklist discharge criteria. (DOCX)

S2 Table High-risk patient PACU discharge checklist. (DOCX)

S3 Table STROBE statement. Checklist of items that should be included in reports of observational studies.
(DOCX)
