# Supplementary figures and images for: The impact of the incorporation of a feasible postoperative mortality model at the Post-Anaesthestic Care Unit (PACU) on postoperative clinical deterioration: A pragmatic trial with 5,353 patients
Source: PLoS One. 2021 Nov 15;16(11):e0257941. doi: 10.1371/journal.pone.0257941 (PMC8592468; doi:10.1371/journal.pone.0257941)

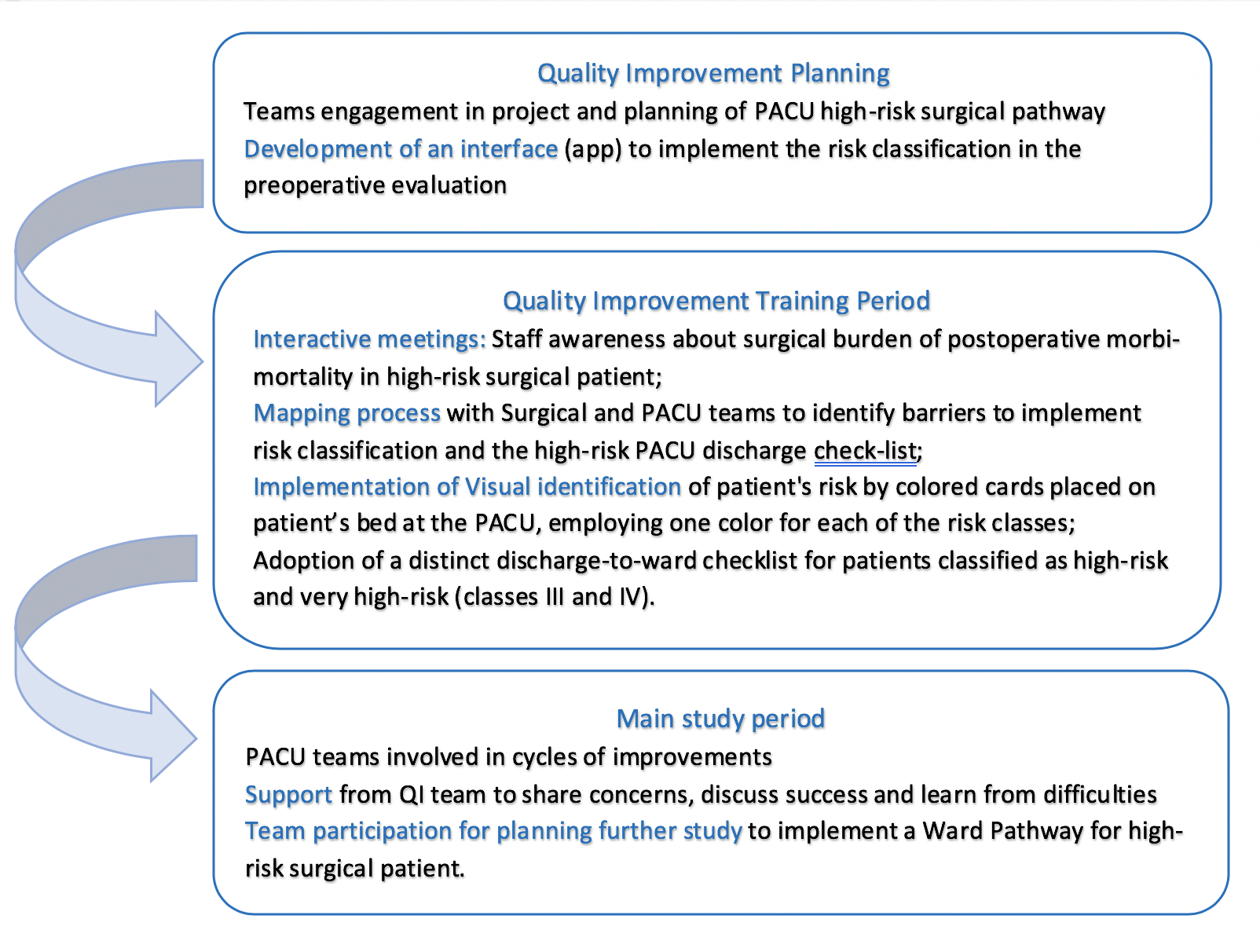

Supplement: S1 Fig — (TIF) [file pone.0257941.s001.tif]

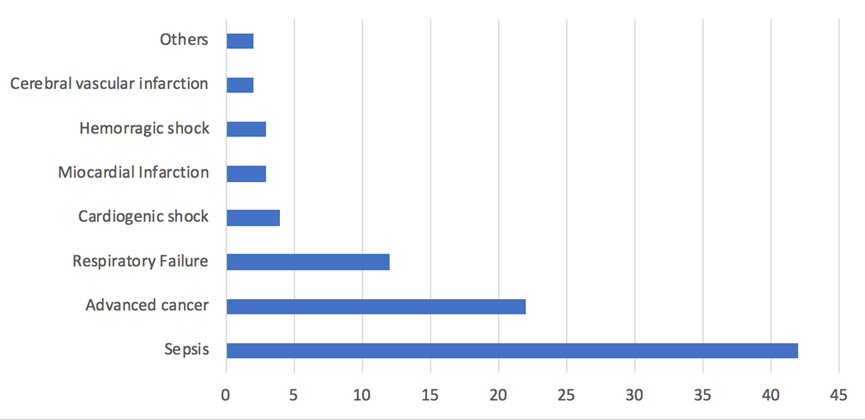

Supplement: S2 Fig — (TIF) [file pone.0257941.s002.tif]

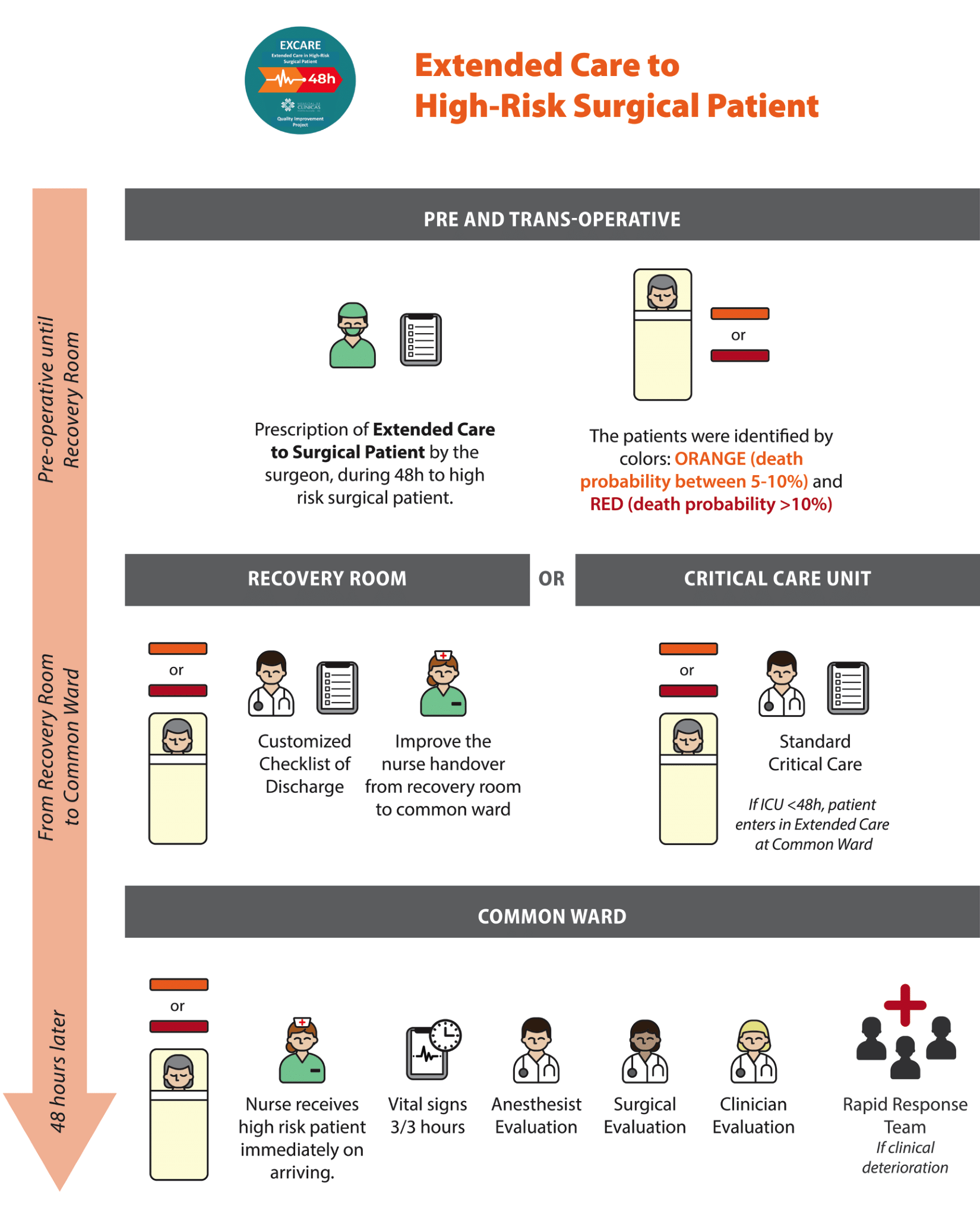

Supplement: S3 Fig — (TIF) [file pone.0257941.s003.tif]
