# Supplementary material for: The impact of the incorporation of a feasible postoperative mortality model at the Post-Anaesthestic Care Unit (PACU) on postoperative clinical deterioration: A pragmatic trial with 5,353 patients
Source: PLoS One. 2021 Nov 15;16(11):e0257941. doi: 10.1371/journal.pone.0257941 (PMC8592468; doi:10.1371/journal.pone.0257941)
Supplement: S1 Table — (DOCX) [file pone.0257941.s004.docx]

**S1 Table.** Standard PACU checklist discharge criteria.

| **Standard PACU discharge checklist**  (all criteria must be present at discharge) |
| --- |
| ( ) Stable vital signs - Hemodynamic stability. Values of blood pressure, heart rate and SPO_2_ must be close to those observed in the preoperative period or at an acceptable level defined by the anaesthesiologist. |
| ( ) Awake or with preoperative sensory pattern - The patient must be recovered from the effect of anaesthetic agents. |
| ( ) SPO_2_ > 90% - The patient must be able to maintain SPO_2_ above 90%, with or without the use of supplemental oxygen therapy. |
| ( ) Pain control - Postoperative pain must be controlled (visual analogue pain scale < 3) |
| ( ) Absence of nausea and vomiting - Postoperative nausea and vomiting should be treated with a multimodal regimen. |
| ( ) Absence of surgical bleeding - Surgical bleeding must be reported to the surgical team |
| ( ) Absence of motor block secondary to regional anaesthesia (or motor block in regression) - The patient must have the absence of motor block or motor block in regression and be able to perform knee flexion (Bromage Scale 1). |

PACU, Post-Anaesthetic Care Unit.
