# Supplementary material for: The impact of the incorporation of a feasible postoperative mortality model at the Post-Anaesthestic Care Unit (PACU) on postoperative clinical deterioration: A pragmatic trial with 5,353 patients
Source: PLoS One. 2021 Nov 15;16(11):e0257941. doi: 10.1371/journal.pone.0257941 (PMC8592468; doi:10.1371/journal.pone.0257941)
Supplement: S3 Table — Checklist of items that should be included in reports of observational studies. (DOCX) [file pone.0257941.s006.docx]

**S6 Table. STROBE Statement—checklist of items that should be included in reports of observational studies**

|  | **Item No** | **Recommendation** | **Insert**  **page #** |
| --- | --- | --- | --- |
| **Title and abstract** | 1 | (*a*) Indicate the study’s design with a commonly used term in the title or the abstract | 1 |
|  |  | (*b*) Provide in the abstract an informative and balanced summary of what was done and what was found | 2 |
| **Introduction** |  |  |  |
| Background/rationale | 2 | Explain the scientific background and rationale for the investigation being reported | 4 |
| Objectives | 3 | State specific objectives, including any prespecified hypotheses | 5 |
| **Methods** |  |  |  |
| Study design | 4 | Present key elements of study design early in the paper | 5 |
| Setting | 5 | Describe the setting, locations, and relevant dates, including periods of recruitment, exposure, follow-up, and data collection | 5,6 |
| Participants | 6 | (*a*) *Cohort study*—Give the eligibility criteria, and the sources and methods of selection of participants. Describe methods of follow-up  *Case-control study*—Give the eligibility criteria, and the sources and methods of case ascertainment and control selection. Give the rationale for the choice of cases and controls  *Cross-sectional study*—Give the eligibility criteria, and the sources and methods of selection of participants | 5,6 |
|  |  | (*b*) *Cohort study*—For matched studies, give matching criteria and number of exposed and unexposed  *Case-control study*—For matched studies, give matching criteria and the number of controls per case | NA |
| Variables | 7 | Clearly define all outcomes, exposures, predictors, potential confounders, and effect modifiers. Give diagnostic criteria, if applicable | 7,8 |
| Data sources/ measurement | 8* | For each variable of interest, give sources of data and details of methods of assessment (measurement). Describe comparability of assessment methods if there is more than one group | 8 |
| Bias | 9 | Describe any efforts to address potential sources of bias |  |
| Study size | 10 | Explain how the study size was arrived at | 8, 9 |
| Quantitative variables | 11 | Explain how quantitative variables were handled in the analyses. If applicable, describe which groupings were chosen and why | 8,9 |
| Statistical methods | 12 | (*a*) Describe all statistical methods, including those used to control for confounding | 8,9 |
|  |  | (*b*) Describe any methods used to examine subgroups and interactions | 8, 9 |
|  |  | (*c*) Explain how missing data were addressed |  |
|  |  | (*d*) *Cohort study*—If applicable, explain how loss to follow-up was addressed  *Case-control study*—If applicable, explain how matching of cases and controls was addressed  *Cross-sectional study*—If applicable, describe analytical methods taking account of sampling strategy | NA |
|  |  | (*e*) Describe any sensitivity analyses | 8,9 |
| **Results** |  |  |  |
| Participants | 13* | (*a*) Report numbers of individuals at each stage of study – eg numbers potentially eligible, examined for eligibility, confirmed eligible, included in the study, completing follow-up, and analysed | 9, 10 |
|  |  | (*b*) Give reasons for non-participation at each stage | 9 |
|  |  | *(c)* Consider use of a flow diagram | 9 |
| Descriptive data | 14***** | *(a)* Give characteristics of study participants (eg demographic, clinical, social) and information on exposures and potential confounders | 10,11 |
|  |  | *(b)* Indicate number of participants with missing data for each variable of interest | NA |
|  |  | *(c) Cohort study* – Summarise follow-up time (eg, average and total amount) | 9 |
| Outcome data | 15* | *Cohort study* – Report numbers of outcome events or summary measures over time | 9,12,13 |
|  |  | *Case-control study*  - Report numbers in each exposure category, or summary measures of exposure | NA |
|  |  | *Cross-sectional study* - Report numbers of outcomes events or summary measures | NA |
| Main results | 16 | *(a)* Give unadjusted estimates and, if applicable, confounder-adjusted estimates and their precision (eg, 95% confidence interval). Make clear which confounders were adjusted for and why they were included | 11, 12 |
|  |  | *(b)* Report category boundaries when continuous variables were categorized | 11, 12 |
|  |  | *(c)* If relevant, consider translating estimates of relative risk and absolute risk for a meaningful time period | NA |
| Other analyses | 17 | Report other analyses done – eg analyses of subgroups and interactions, and sensitivity analyses |  |
| **Discussion** |  |  |  |
| Key results | 18 | Summarise key results with reference to study objectives | 15 |
| Limitations | 19 | Discuss limitations of the study, taking into account sources of potential bias or imprecision. Discuss both direction and magnitude of any potential bias | 16 |
| Interpretation | 20 | Give a cautious overall interpretation of results considering objectives, limitations, multiplicity of analyses, results from similar studies, and other relevant incidence | 15-17 |
| Generalisability | 21 | Discuss the generalisability (external validity) of the study results | 17,18 |
| **Other information** |  |  |  |
| Funding | 22 | Give the source of funding and the role of the funders for the present study and, if applicable, for the original study on which the present article is based | 19 |

*Give information separately for cases and controls in case-control studies and, if applicable, for exposed and unexposed groups in cohort and cross-sectional studies.
